# LC-MS/MS Method for Determination of Hydroxychloroquine and Metabolites: Application in a Pharmacokinetic Study

**DOI:** 10.1155/2022/6058445

**Published:** 2022-01-05

**Authors:** Lili Cui, Zhipeng Wang, Shi Qiu, Mengwei Zhang, Yanping Liu, Fengjing Xu, Xinhua Song, Shouhong Gao, Wansheng Chen

**Affiliations:** ^1^Department of Pharmacy, Second Affiliated Hospital of Naval Medical University, Shanghai 200003, China; ^2^Research and Development Center of Chinese Medicine Resources and Biotechnology, Institute of Chinese Materia Medica, Shanghai University of Traditional Chinese Medicine, Shanghai 201203, China; ^3^Chemistry and Biological Engineering College, Yichun University, Yichun 336000, China; ^4^College of Traditional Chinese Medicine, Yunnan University of Traditional Chinese Medicine, Kunming, Yunnan 650500, China

## Abstract

Hydroxychloroquine (HCQ) was originally used as an antimalarial and immunomodulation drug. We developed and validated a simple and sensitive ultrahigh performance liquid chromatography-tandem mass spectrometry (UHPLC-MS/MS) method for simultaneous quantitation of HCQ and its three metabolites in rat blood, and reported their pharmacokinetic parameters. The chromatographic separation and detection of analytes were achieved within 4 min on ZORBAX SB-C_8_ (3.5 *μ*m, 2.1 × 150 mm) column with gradient elution, and the flow rate was 0.25 mL/min. Simple protein precipitation was successfully applied for sample pretreatment. The HCQ displays a good linearity in the range of 2.0–5000.0 ng/mL, and the three metabolites also show good linearity ranging from 1.0 to 2500.0 ng/mL, with all correlation coefficients (*R*^2^) better than 0.98. In conclusion, this rapid, sensitive method was successfully developed, validated, and then applied to a pharmacokinetic study of HCQ in rat model in high dose. The results of the pharmacokinetic study presented an average half-life time 21.14 ± 10.31 h (mean ± SD) of HCQ, which is much shorter in human compared to that in mice. For the three metabolites, longer half-life times (approximately 100 h) were shown in rat.

## 1. Introduction

Hydroxychloroquine (HCQ), an antimalarial drug, is the hydroxyl-substituted product of chloroquine (CQ), which has become the backstone in the treatment of rheumatic arthritis (RA) and systemic lupus erythematosus (SLE) in recent years because of its characteristics of immunomodulatory, hypolipidemic, antithrombotic effect, and, furthermore, the HCQ was utilized to reduce the risk of malignant tumors and treat sarcoidosis and still disease [1].

Recent *in vitro* studies had confirmed that HCQ and CQ have antiviral activity against SARS-CoV-2 virus and that the efficacy of HCQ is better than that of CQ (EC_50_, 0.72 vs 5.47 *μ*mol/L), but many clinical studies had reported that HCQ was ineffective in human body for Covid-19 [2–10]. In these studies, the HCQ was administered in different doses (200–1200 mg) and in different frequencies (once a day to three times a day) for several days (4–21 days), and these regimens (high dose and multiple administrations) differ greatly from the HCQ prescription in SLE and RA treatment. HCQ is metabolized into three active metabolites, that is, bisdesethylchloroquine (BDCQ), desethylchloroquine (DCQ), and desethylhydroxychloroquine (DHCQ) [11] in the liver by CYP 450 enzymes. The CYP 450 enzymes play critical roles in the catabolism of HCQ, which are mainly mediated by some subtypes such as CYP3A4, CYP3A5, CYP2D6, and CYP2C8 and the gene polymorphisms of them also affect the blood concentrations of HCQ and three metabolites [12]. In a study, the pharmacokinetic parameters after a single oral administration of 200 mg HCQ in 20 healthy Chinese men were reported, and the results showed that the *C*_max_ was 44.1 ± 27.6 ng/mL (mean ± SD), *t*_max_ was 3.85 ± 1.04 h, AUC_0–60_ was 1789 ± 383 ng h/mL, and *t*_1/2_ was about 298 ± 105 h. The HCQ showed an extremely slow elimination in human [12]. In comparison, Chhonker et al. [13]. reported the pharmacokinetic parameters of HCQ after intravenous injection of 5 mg/kg HCQ in mice: *t*_1/2_ 12.7 ± 1.1 h, AUC_0–∞_ 5577.8 ± 881.8 ng h/mL, and AUC_0–72_ 5490.6 ± 890.0 ng h/mL. The half-life time of HCQ in mice is much shorter than that in human. These results make the therapeutic drug monitoring and pharmacokinetic study of high-dose HCQ necessary.

Some studies have reported several methods about quantification of the HCQ and its metabolites based on liquid chromatography-tandem mass spectrometry (LC-MS/MS) in recent 5 years, and their applications in quantifying the HCQ and its metabolites in human blood and mouse blood and tissues [11, 13–16]. However, most of these methods have compromised to narrow calibration range, complex sample pretreatment, and/or chromatographic separation or not including the metabolites. In addition, the pharmacokinetic characteristics of HCQ have been reported in human and mouse, but the metabolic pattern of HCQ in rat has not been reported, especially in a dose used in Covid-19. Therefore, this study was designed to establish a simple, rapid, and sensitive method for simultaneous determination of HCQ and its three metabolites in rat blood by LC-MS/MS, and to explore the pharmacokinetic characteristics of HCQ in rats in a Covid-19 dose.

## 2. Materials and Methods

### 2.1. Chemicals and Reagents

The standards including BDCQ (Lot: 7-MJC-76-1), DCQ (Lot: 3-NZZ-137-6), DHCQ (Lot: 6-MR-3-1), and HCQ (Lot: X11J11G109865) were purchased from Toronto Research Chemicals (Toronto, Canada). HCQ-d4 (Lot: ZZS-20-040-B5) was used as the internal standard (IS) for all the analytes and supplied by Shanghai Zhenzhun Biotechnology Co., Ltd. (Shanghai, China). HPLC-grade methanol (MeOH) was obtained from Merck (Merck Company, Darmstadt, Germany). HPLC-grade formic acid (FA) and ammonium acetate were purchased from Tedia Company Inc. (Tedia, Fairfield, OH, USA). Double-distilled water was obtained from the A.S. Watson Group (Hong Kong, China) Ltd. and was used throughout. All other reagents used in the study were procured from qualified chemicals suppliers and of analytical grade. Blank blood was collected with heparin-anticoagulation in employing rat as an experimental model in the Animal Experiment Center of Shanghai University (Shanghai, China), and stored at −80°C until use.

### 2.2. LC-MS/MS Instrumentation

An Agilent 1290 ultrahigh performance liquid chromatography coupled to 6460A mass spectrometer, which was equipped with a binary pump (G4220A), online degasser (G1969-80230), an autosampler (G4226A), and column oven (G1316C), was used in our study (Agilent Technologies, Santa Clara, CA, USA). All data were acquired and processed using Agilent Masshunter data processing software (version B.06.00; Agilent Technologies).

### 2.3. Liquid Chromatographic Conditions

The chromatographic separation of four analytes was achieved based on a ZORBAX SB-C_8_ column (3.5 *μ*m, 2.1 × 150 mm; Agilent Technologies) at a flow rate of 0.25 mL/min with column temperature set at 40°C. The mobile phase A was 0.2% FA plus 10 mmol/L ammonium acetate in water, and mobile phase B was MeOH. The initial mobile phase consisted 95% of phase A and 5% phase B. Gradient variation was as follows: 0–1 min, 95% phase A; 1–1.1 min 95% ⟶ 5% phase A; and maintained 5% phase A until 4 min. The injection volume of sample was 5 *μ*L with a 10-second needle wash using 75% MeOH aqueous solution.

### 2.4. Mass Spectrometry Conditions

The mass spectrometry detection was achieved on an Agilent 6460A mass spectrometer equipped with Agilent jet stream electrospray ionization (AJS-ESI) source. Data acquisition was operated in the multiple reaction monitoring (MRM) mode. The optimized mass spectrometer source settings were utilized: capillary voltage 4500 V, sheath gas temperature 400°C, sheath gas flow 12 L/min, nebulizer pressure 45 psi, dry gas temperature 320°C, and dry gas flow 10 L/min. All analytes were detected in positive ionization mode. The optimized MRM parameters for HCQ and its three metabolites are shown in Table 1. The peak widths of precursors and product ions were maintained at 0.7 amu at half-height of peak, and the dwell time for all analytes was 100 ms.

### 2.5. Preparation of Standard and Quality Control (QC) Samples

The stock solutions of HCQ and its metabolites BDCQ, DCQ, DHCQ as well as the IS HCQ-d4 were individually prepared in MeOH aqueous solution (50 : 50, V : V), and 2.01, 2.01, 2.02, 2.00, 2.01, and 1.0 mg of HCQ, BDCQ, DCQ, DHCQ, and HCQ-d4 were accurately weighed and prepared, respectively. The final concentrations of five stock solutions were all at 1.0 mg/mL. All stock solutions were aliquoted and stored at −80°C. The stock solutions of all analytes were further diluted with 10% MeOH and combined to prepare the calibration standards and quality control samples (QCs), and 25 *μ*L of combined working solutions was added to 475 *μ*L rat blood to obtain the calibration standards at 2.0, 5.0, 10.0, 20.0, 50.0, 100.0, 200.0, 500.0, 1000.0, 2000.0, 4000.0, and 5000.0 ng/mL for HCQ; 1.0, 2.5, 5.0, 10.0, 25.0, 50.0, 100.0, 250.0, 500.0, 1000.0, 2000.0, and 2500.0 ng/mL for BDCQ, DCQ, and DHCQ. QCs were separately weighed and prepared using the same way at three different concentration levels including the low quality control (LQC) (5.0 ng/mL for HCQ and 2.5 ng/mL for three metabolites), middle quality control (MQC) (2000.0 ng/mL for HCQ and 1000.0 ng/mL for three metabolites), and high quality control (4000 ng/mL for HCQ and 2000.0 ng/mL for three metabolites).

### 2.6. Blood Sample Pretreatment

For the blood sample, the pretreatment was performed based on one-step protein precipitation. Briefly, 50 *μ*L sample was transferred into a 1.5 mL polypropylene tube and spiked with 200 *μ*L of acetonitrile (containing 100 ng/mL HCQ-d4). The mixture was vigorously vortex mixed for 3 minutes prior to centrifugation at 14500 × *g* for 10 min at room temperature, and 5 *μ*L of the supernatant was injected directly into the LC-MS/MS system for analysis.

### 2.7. Method Validation

This newly developed LC-MS/MS method was fully validated according to the guidance of FDA and Chinese Pharmacopoeia (the 2015 edition).

Method validation, including selectivity, matrix effect and recovery, linearity, interday and intraday precision and accuracy, and stability, was carried out using the same way reported before [17, 18].

### 2.8. Animals Experiment

The protocol of the animal study was approved by the Experimental Animal Ethics Committee of the Naval Medical University. Healthy male SD (Sprague–Dawley) rats, 200–220 g, were obtained from the animal experiment center of Shanghai University and were fed with standard food and water for 1 week before the experiment. Pharmacokinetic study of HCQ was conducted in rats after an overnight food fasting (12 h) with free access to water. The animal study was carried out in accordance with the National Institutes of Health Guide for the Care and Use of Laboratory animals.

In this study, SD rats were dosed with 36 mg/kg HCQ intragastrically. The dose of HCQ in this experiment was calculated according to HCQ concentrations in a longitudinal cohort analysis of SLE [19] and a HCQ exposure monitoring experiment conducted in Covid-19 treatment centers at Shanghai, China (data not shown). Five male SD rats were fed with HCQ suspension (36 mg/kg, 0.5% CMC-Na) at 8 : 00 am in the morning, and approximately 300 *μ*L of blood was collected at 0, 0.083, 0.25, 0.5, 0.75, 1, 2, 4, 6, 8, 12, 24, 48, 72, and 96  h into heparin sodium-pretreated tubes. The samples were gently mixed and then stored at −80°C until analysis.

The pharmacokinetic parameters of HCQ and its three metabolites in blood were calculated using a noncompartmental model with Drug and Statistics (DAS) software (version 2.0; China Pharmacological Society). The weighing factor was designated as 1/*C*^2^ for all analytes.

## 3. Results and Discussion

### 3.1. Optimization of Chromatographic and MS/MS Conditions

**Figure 1:**
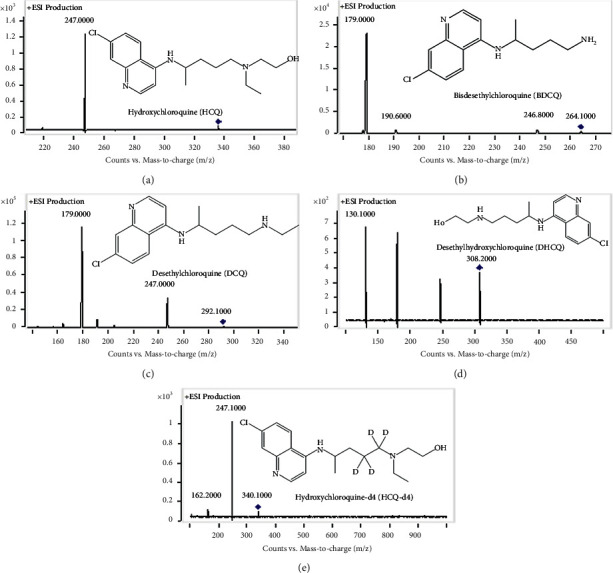
Chemical structures and full scan in product ion mode for HCQ (a), BDCQ (b), DCQ (c), DHCQ (d), and IS (e).

To obtain the optimal peak resolution, reproducibility and shorter chromatographic separation time, lots of chromatographic conditions, including different analytical columns, mobile phases, and elution procedures, were tested in the method development process. First, the effects of different chromatographic columns on the retention and separation of four analytes compounds were investigated. We tested Agilent ZORBAX SB-C_18_ (2.1 mm × 100 mm, 3.5 *μ*m), Agilent ZORBAX SB-C_8_ (2.1 × 150 mm, 3.5 *μ*m), Waters Xbridge®HILIC (2.1 mm × 100 mm, 3.5 *μ*m), Waters Atlantis T3 (2.1 mm × 100 mm, 3 *μ*m), Agilent Eclipse XDB-C_18_ (2.1 mm × 150 mm, 3.5 *μ*m), Waters Xbridge C_18_(2.1 mm × 50 mm, 3.5 *μ*m), Waters XSELECT CSH C_18_ (2.1 mm × 50 mm, 2.5 *μ*m), and some other analytical columns. It was found that the HCQ and metabolites were well retained and separated on the Agilent ZORBAX SB-C_8_ column, and the analytical time was relatively shorter among all the tested columns. Second, the effects of different mobile phases and additives were investigated, and different concentrations of FA, ammonium acetate and their mixed solutions in MeOH, acetonitrile or/and water, and acidified MeOH and acetonitrile were tried successively. When FA or ammonium acetate aqueous solution was used alone, the chromatographic peak shape was seriously tailing. Acidified MeOH or acetonitrile could not further improve the peak shape and a poor retention was shown. According to the principle of simplicity and rapidity, 0.2% FA plus 10 mmol ammonium acetate aqueous solution (phase A) and MeOH (phase B) were finally chosen as the optimal mobile phases. Lastly, the gradient elution procedure and the mass spectrometry detection parameters such as collision energy, fragmentor energy, nebulizer pressure, and drying gas were optimized. Combined with C_8_ column, all the analytes presented symmetrical peak shape and appropriate retention time.  Figure 1 shows the fragment structures in the product scan mode of four analytes and IS.

### 3.2. Sample Pretreatment

Matrix interferents removement is the critical step in the pretreatment of biological samples, which is the base of high and stable recovery and matrix effect. In the pretreatment method development, we tested a variety of methods to remove proteins and other potential interfering substances in blood. The most common biological sample pretreatment methods are protein precipitation, liquid-liquid extraction, and solid phase extraction [20]. In the first step, the protein precipitation was carried out using organic solvents such as MeOH, acetonitrile, acidified MeOH, acidified acetonitrile, and their mixtures. The acidification of MeOH or acetonitrile actually yielded a low recovery for all the analytes (<40%), which might be explained by the higher water solubility of HCQ and its metabolites in acid solutions. The MeOH and acetonitrile, however, showed high recovery and steady matrix effect. To explore a better extraction method, we still tried solid phase extraction and Sartorius' ultrafiltration centrifuge tube for biological sample pretreatment. The results indicated that all the analytes might be adsorbed in the stationary phase or plastic surface, which resulted in extremely low recovery (<20%). During liquid-liquid extraction, it was found that the extraction recovery of the four compounds was low (<50%), and the matrix effect was strong and unsteady (RSD% > 15%). To sum up, 200 *μ*L acetonitrile was used to eliminate the possible interferences in 100 *μ*L blood sample by protein precipitation, and the highest (>86%) and consistent extraction recovery of all the analyte was achieved. Compared with solid phase extraction and liquid-liquid extraction, this protein precipitation method is fast, simple, and economical.

### 3.3. Method Validation

#### 3.3.1. Selectivity

**Figure 2:**
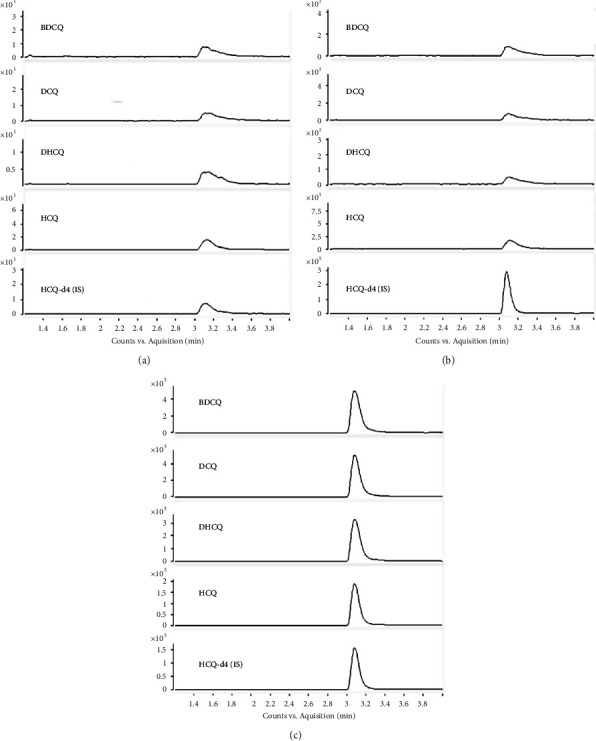
Typical MRM chromatograms of (a) blank rat blood; (b) blank blood spiked with 80 ng/mL IS; (c) real rat blood sample.

In order to evaluate the selectivity, we compared the responses from blank, IS spiked, and real samples (Figure 2), and the results proved that there were no significant interferences observed in corresponding retention times of the analytes and IS as the responses in blank sample were not more than 20% of the four analytes in the LLOQ sample and 5% of IS.

#### 3.3.2. Matrix Effect and Extraction Recovery

The evaluation of matrix effects in biological samples for quantitative analysis of drugs by mass spectrometry is an important aspect of method validation [17]. The range of extraction recovery and matrix effect for all analytes was calculated using the LQC and HQC concentrations in six replicates and the results are 86.42–93.77% and 66.20–87.98% for recovery and matrix effect, respectively, with their RSD% all less than 15%. Protein precipitation using acetonitrile obtained a high and consistent extraction recovery and the interference was purified to a great extent. The detailed results of matrix effects and extraction recovery are shown in Table 2.

#### 3.3.3. Linearity of Calibration Curves

The calibration curve was constructed by calculating the peak area ratio (analyte/IS) of the calibration standards to the measured concentrations. Twelve calibration standards were obtained from the spiked samples, and the best linear and least square residuals were obtained when the weighing factor was 1/*χ*^2^. The linear correlation coefficients of all analytes are greater than 0.98. The typical regression equations of the standard curve are shown in Table 3. The RE% is within ±15% (within ±20% for the LLOQ) of the back-calculation deviations of all calibration standards, which are in line with the criteria.

#### 3.3.4. Interday and Intraday Precision and Accuracy

The QC samples in four concentration levels (LLOQ, low, middle, and high) and LLOQ samples were assessed in five replicates at three separate analytical lots to determine the intraday and interday precision and accuracy. The results of interdayand intraday precision and accuracy are acceptable and are summarized in Table 4.

#### 3.3.5. Stability

We investigated the stability of four analytes in rat blood at two concentration levels (low and high). As a consequence, the analytes were steady in autosampler for 24 h with deviations located within ±15% (RE%). After being stored at −80°C for 30 days, there are no significant deviations in QC samples. Meanwhile, no obvious deviations were found after three freeze-thaw cycles. Bench-top stability also presented deviations within ±15% (data not shown). All the results are shown in Table 5.

#### 3.3.6. Application in HCQ Pharmacokinetic Study

**Figure 3:**
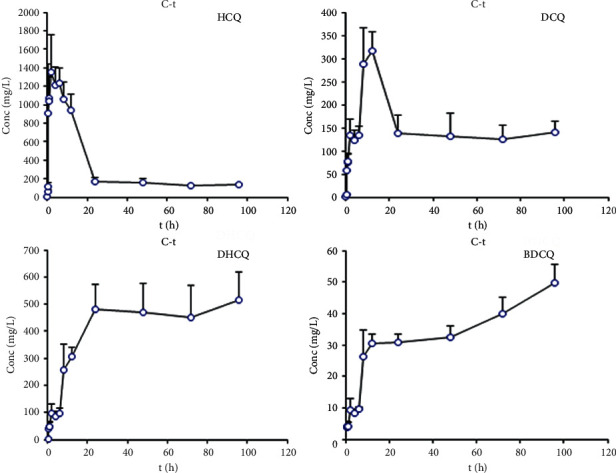
The mean concentration-time(C-t) curves of HCQ and three metabolites in rat blood after intragastric administration.

An UHPLC-MS/MS method was established and validated for determining HCQ and its three metabolites, which was then applied to a pharmacokinetic study of HCQ. HCQ (36 mg/kg) was given to rats by intragastric administration. The blood concentration-time curves of HCQ and its three metabolites are shown in Figure 3. The pharmacokinetic parameters of HCQ and three metabolites in rats are shown in Table 6. T*C*_max_ of HCQ in rats was 1440.72 ± 298.24 *μ*g/L (mean ± SD) and *T*_1/2_ was 21.14 ± 10.31 h. AUC_0–∞_ was 42774.94 ± 8495.26 *μ*g/L*∗*h, and the clearance rate was 1.52 ± 0.38 L/h/kg. The pharmacokinetic parameters of HCQ in rats were compared with that in mice reported in literature, and the blood elimination *T*_1/2_ and AUC_0–∞_ were similar to that in mice, while the *C*_max_ in rats was 40 times higher than that of mice and these results may be attributed largely to a higher administration dose [13]. In human, the *T*_1/2_ of HCQ was much longer and *C*_max_ of HCQ in rats was approximately 30 times higher than that in human [11], and the clearance rate was higher than that in human body, which showed a big difference in HCQ metabolism between human and animal model. For the three metabolites, longer average half-life times (more than 100 h) were found, in addition, the DHCQ showed the highest AUC and *C*_max_ values than the DCQ and BDCQ. In a study, the association of gene polymorphisms of CYP 2D6 and blood HCQ level was assessed in SLE patients, and the results showed that the CYP 2D6 polymorphism was significantly associated with the DHCQ/HCQ ratio, and this may explain why there is a wide variation of HCQ concentration [21]. However, in this rat study, the gene polymorphisms of CYP enzymes were not determined, and there are wide variations of pharmacokinetic parameters of HCQ and its three metabolites among rats, which may indicate different expression levels or activities of CYP enzymes in rats.

The in vivo exposure of drug had a close relationship with the therapeutic results, and concentrations located in the therapeutic window can obviously increase the responses and decrease the adverse reactions. A study investigated the concentration-response relationship of HCQ in the treatment of RA, and different doses (400, 800, or 1,200 mg HCQ daily) were prescribed and the blood exposure of HCQ was proven to be positively associated with the gastrointestinal adverse events, in addition, the blood concentration of DHCQ had a positive correlation with response in RA patients (*P* < 0.001) while the BDCQ was believed to be associated with the ocular adverse events (*P*=0.036) [22], and this may be explained by the different in vivo exposure of metabolites. In patients with cutaneous lupus erythematosus, a higher blood concentration of HCQ was associated with complete remission (910 ng/mL, mean value) compared with a partial remission (692 ng/mL, mean value) and treatment failure (569 ng/mL, mean value) (*P*=0.007) [23]. These results demonstrated that monitoring of HCQ is necessary for HCQ dose optimization. In our study, the metabolism features of high-dose HCQ in rat were reported, and further studies in exploring the tissue distribution of HCQ in rat organs/tissues, especially in high-dose and long-term regimen, are necessary. Combining the pharmacokinetic parameters of HCQ and the organs/tissue distribution may be helpful in clarifying the efficacy and adverse effect of HCQ in a drug metabolism aspect.

## 4. Conclusion

A simple, rapid, sensitive, and reproducible UHPLC-MS/MS method was developed and validated in this study, which was suitable for simultaneous determination of HCQ and its three metabolites (BDCQ, DHCQ, and DCQ) in rat blood. After optimizing the chromatographic separation and mass spectrometry detection conditions, a shorter analytical time (4 min) and lower LLOQ (approximately 1.0 ng/mL) for all analytes were achieved. In addition, the pharmacokinetic parameters of high-dose HCQ and its three metabolites in rats were firstly reported in this study. The metabolic pattern of HCQ is comparable to that in mouse and is significantly different from that in human.

## Figures and Tables

**Table 1:** Optimized MRM parameters for the detection of four analytes and IS.

Analytes	Precursor ion (*m*/*z*)	Product ion (*m*/*z*)	Fragmentor (*V*)	Collision energy (eV)	Ionization mode

BDCQ	264.1	179	120	24	Positive
DCQ	292.1	179	85	23	Positive
DHCQ	308.2	130.1	70	17	Positive
HCQ	336.1	247	110	18	Positive
HCQ-d4 (IS)	340.1	247.1	90	20	Positive

**Table 2:** Extraction recovery and matrix effect data of the analytes in rat blood (*n* = 6).

Analytes	Nominal concentration (ng/mL)	Extraction recovery		Matrix effect	
		Mean ± SD	RSD (%)	Mean ± SD	RSD (%)

BDCQ	2.5	90.27 ± 12.97	14.37	66.20 ± 3.84	5.81
	2000	86.64 ± 5.45	6.29	83.14 ± 2.58	3.10

DCQ	2.5	89.59 ± 10.98	12.26	75.87 ± 4.87	6.42
	2000	87.14 ± 4.06	4.66	85.15 ± 1.76	2.06

DHCQ	2.5	86.42 ± 12.24	14.16	77.37 ± 3.26	4.21
	2000	92.00 ± 3.67	3.99	87.13 ± 1.83	2.10

HCQ	5	92.14 ± 9.86	10.70	73.56 ± 2.79	3.79
	4000	93.77 ± 4.28	4.56	87.98 ± 1.44	1.64

**Table 3:** Linear regression equation, range, and correlation coefficients of four analytes.

Analytes	Regression equations	Calibration range (ng/mL)	*r* ^2^

BDCQ	*y*=0.181*∗x*+0.006	1.00∼2500.00	0.987
DCQ	*y*=0.134*∗x*+0.005	1.00∼2500.00	0.994
DHCQ	*y*=0.110*∗x*+0.003	1.00∼2500.00	0.990
HCQ	*y*=0.216*∗x*+0.009	2.00∼5000.00	0.990

**Table 4:** Interday and intraday precision and accuracy of four analytes in rat blood (*n* = 5).

Analytes	Nominal concentration (ng/mL)	Intraday (*n* = 5)			Interday (*n* = 5)		
		Measured concentration (mean ± SD, ng/mL)	Precision (RSD%)	Accuracy (RE%)	Measured concentration (mean ± SD, ng/mL)	Precision (RSD%)	Accuracy (RE%)

BDCQ	1	1.03 ± 0.005	0.56	3.98	1.02 ± 0.02	2.59	2.45
	2.5	2.65 ± 0.05	1.99	6.34	2.54 ± 0.15	6.24	1.82
	1000	1085.1 ± 14.4	1.33	8.51	1097.3 ± 40.1	3.66	9.73
	2000	1797.67 ± 91.65	5.10	−10.11	1758.68 ± 59.67	3.39	−12.06

DCQ	1	1.04 ± 0.02	2.43	4.26	1.03 ± 0.04	4.15	3.49
	2.5	2.57 ± 0.07	2.80	3.19	2.55 ± 0.08	3.24	2.23
	1000	1057.50 ± 86.3	8.17	5.75	1044.80 ± 81.4	7.80	4.48
	2000	2029.26 ± 157.29	7.75	1.46	2033.73 ± 138.43	6.81	1.68

DHCQ	1	1.04 ± 0.03	3.37	4.83	1.02 ± 0.03	3.45	2.39
	2.5	2.61 ± 0.09	3.77	4.67	2.53 ± 0.20	8.15	1.34
	1000	1096.61 ± 56.7	5.18	6.69	1097.0 ± 58.9	5.37	9.70
	2000	1815.69 ± 23.66	1.30	−9.21	1796.74 ± 33.65	1.87	−10.16

HCQ	2	2.02 ± 0.02	1.21	1.20	2.06 ± 0.06	3.13	3.02
	5	5.14 ± 0.10	2.11	2.94	5.05 ± 0.20	4.14	1.11
	2000	2040.6 ± 175.1	8.59	2.04	2022.1 ± 158.1	7.82	1.10
	4000	4276.65 ± 368.71	8.62	6.91	4262.63 ± 321.74	7.55	6.56

**Table 5:** Stability of analytes in different conditions (*n* = 5).

Analytes	Nominal concentration (ng/mL)	Short-term stability (6 h)		Long-term stability (30 day at −80°C)		Freeze-thaw stability (3 cycles)	
		Measured concentration (mean ± SD, ng/mL)	RSD (%)	Measured concentration (Mean ± SD, ng/mL)	RSD (%)	Measured concentration (Mean ± SD, ng/mL)	RSD (%)

BDCQ	2.5	2.61 ± 0.05	2.13	2.54 ± 0.04	1.80	2.59 ± 0.04	1.71
	2000	1842.63 ± 48.07	2.61	1765.50 ± 32.09	1.82	1733.13 ± 29.97	1.73

DCQ	2.5	2.63 ± 0.07	2.89	2.55 ± 0.03	1.42	2.57 ± 0.04	1.61
	2000	2098.46 ± 164.60	7.84	2142.71 ± 148.78	6.94	1943.91 ± 77.36	3.98

DHCQ	2.5	2.60 ± 0.05	2.12	2.48 ± 0.05	2.22	2.63 ± 0.06	2.40
	2000	1859.79 ± 70.66	3.80	1876.02 ± 88.45	4.71	1753.78 ± 69.66	3.97

HCQ	5	5.31 ± 0.14	2.67	5.08 ± 0.10	2.15	5.12 ± 0.06	1.24
	4000	4395.41 ± 243.53	5.54	4170.81 ± 313.74	7.52	4362.96 ± 325.76	7.47

**Table 6:** Blood pharmacokinetic parameters of HCQ and its three metabolites in rat (*n* = 5).

Parameters	HCQ	DCQ	DHCQ	BDCQ
*T*_1/2_ (h)	21.14 ± 10.31	108.63 ± 82.06	109.82 ± 46.38	110.98 ± 43.54
CL (L/h/kg)	1.52 ± 0.38	1.24 ± 0.54	0.32 ± 0.07	3.39 ± 0.38
AUC_0 ⟶ t_ (*μ*g/)	30515.35 ± 3038.99	14464.13 ± 2068.53	40723.45 ± 5804.73	3257.60 ± 234.57
AUC_0 ⟶ ∞_ (*μ*g/)	42774.94 ± 8495.26	34880.13 ± 17962.93	118353.55 ± 27515.19	10744.56 ± 1248.49
*T*_max_ (h)	4.00 ± 2.83	10.40 ± 2.20	72.00 ± 33.94	96.00 ± 0.00
*C*_max_ (*μ*g/L)	1440.72 ± 298.24	331.83 ± 49.45	551.40 ± 83.66	49.60 ± 6.11

## Data Availability

The methodology and pharmacokinetic data used to support the findings of this study are included in the article.
